# Artificial intelligence in prediction of mental health disorders induced by the COVID-19 pandemic among health care workers

**DOI:** 10.3325/cmj.2020.61.279

**Published:** 2020-06

**Authors:** Krešimir Ćosić, Siniša Popović, Marko Šarlija, Ivan Kesedžić, Tanja Jovanovic

**Affiliations:** 1 Laboratory for Interactive Simulation Systems, Faculty of Electrical Engineering and Computing, University of Zagreb, Zagreb, Croatia *ivan.kesedzic@fer.hr*; 2 Department of Psychiatry and Behavioral Neurosciences, Wayne State University, Detroit, MI, United States of America

## Abstract

The coronavirus disease 2019 (COVID-19) pandemic and its immediate aftermath present a serious threat to the mental health of health care workers (HCWs), who may develop elevated rates of anxiety, depression, posttraumatic stress disorder, or even suicidal behaviors. Therefore, the aim of this article is to address the problem of prevention of HCWs’ mental health disorders by early prediction of individuals at a higher risk of later chronic mental health disorders due to high distress during the COVID-19 pandemic. The article proposes a methodology for prediction of mental health disorders induced by the pandemic, which includes: Phase 1) objective assessment of the intensity of HCWs’ stressor exposure, based on information retrieved from hospital archives and clinical records; Phase 2) subjective self-report assessment of stress during the COVID-19 pandemic experienced by HCWs and their relevant psychological traits; Phase 3) design and development of appropriate multimodal stimulation paradigms to optimally elicit specific neuro-physiological reactions; Phase 4) objective measurement and computation of relevant neuro-physiological predictor features based on HCWs’ reactions; and Phase 5) statistical and machine learning analysis of highly heterogeneous data sets obtained in previous phases. The proposed methodology aims to expand traditionally used subjective self-report predictors of mental health disorders with more objective metrics, which is aligned with the recent literature related to predictive modeling based on artificial intelligence. This approach is generally applicable to all those exposed to high levels of stress during the COVID-19 pandemic and might assist mental health practitioners to make diagnoses more quickly and accurately.

The coronavirus disease 2019 (COVID-19) pandemic and its immediate aftermath present a serious threat to the mental health of health care workers (HCWs), who may develop elevated rates of anxiety, depression, posttraumatic stress disorder (PTSD), or even suicidal behaviors (1). Recent research related to the COVID-19 pandemic (2,3) and 2015 Middle East respiratory syndrome (MERS) outbreak (4) recognizes that HCWs are at high risk for mental illness. Therefore, urgent monitoring of their mental health is needed, particularly early prediction and proper treatments of nurses and physicians who were exposed to a high level of distress by working directly with ill or quarantined persons (5). Mental health risks of highly distressed individuals are further increased when they exhibit low overall stress resilience and have other vulnerability factors, such as the general propensity to psychological distress (6) and low self-control (7). Recognition and identification of such individuals in early stages of acute stress is extremely important in order to prevent the development of more serious long-term mental health disorders, such as PTSD, depression, and suicidal behavior. However, mental disorders are difficult to diagnose, and even more difficult to predict due to the current lack of biomarkers (8) and humans’ subjectivity, as well as unique personalized characteristics of illness that may not be observable by mental health practitioners. Currently, the diagnosis of mental health disorders is mainly based on the symptoms categorized according to the Diagnostic and Statistical Manual of Mental Disorders (DSM-5) (9).

In such circumstances, one of the greatest impacts of digital psychiatry, particularly applied artificial intelligence (AI) and machine learning (ML) (10-15) during the ongoing COVID-19 pandemic, is their ability of early detection and prediction of HCWs’ mental health deterioration, which can lead to chronic mental health disorders. Furthermore, AI-based psychiatry may help mental health practitioners redefine mental illnesses more objectively than is currently done by DSM-5 (14). Regardless of the specific application, ie, prediction, prevention, or diagnosis, AI-based technologies in psychiatry rely on the identification of specific patterns within highly heterogeneous multimodal sets of data (13). These big data sets may include various psychometric scales or mood rating scales, brain imaging data, genomics, blood biomarkers, data based on novel monitoring systems (eg, smartphones), data scraped from social media platforms (16), speech and language data, facial data, dynamics of the oculometric system, attention assessment based on eye-gaze data, as well as various features based on the analysis of peripheral physiological signals (8,17), eg, respiratory sinus arrhythmia, startle reactivity etc. Such AI systems based on multimodal neuro-psycho-physiological features can detect mental health disorders early enough to prevent and reduce the emergence of severe mental illnesses and improve the overall mental health. Therefore, AI has the transformational power to change a subjective diagnostic system in psychiatry to a more objective medical discipline. Also, a new generation of AI in psychiatry might act as a self-explanatory digital assistant to psychiatrists. Definitely, psychiatry today could benefit from AI’s ability to analyze data and recognize patterns and hidden warning signs that a psychotherapist might miss. Such timely information enables making diagnoses more quickly and accurately, and might be lifesaving particularly for all of those HCWs who might have suicidal ideation (18,19) due to heavy mental distress during the COVID-19 pandemic.

Hence, the aim of this article is to address the problem of prevention of HCWs’ mental health disorders by early prediction of individuals who may have a higher risk of later chronic mental health disorders due to high distress during the COVID-19 pandemic. In order to reach this aim and enhance traditional subjective diagnostics and risk assessment approaches, the methodology proposed in this article is based on our extensive experimental research on the selection of resilient candidates for special forces during Survival, Evasion, Resistance and Escape (S.E.R.E.) training in collaboration with Emory University School of Medicine, Atlanta, United States, and Hadassah Hebrew University Hospital, Jerusalem, Israel (20). Similar methodology has been applied in our project related to the selection of resilient candidates for air traffic controllers in cooperation with Harvard Medical School & Massachusetts General Hospital and Croatia Air Traffic Control (17,21). These multi-year experimental research projects are based on a variety of questionnaires and experimental measurements, which include a set of comprehensive multimodal stimuli, corresponding multimodal neuro-physiological, oculometric and acoustic/speech responses, and complex feature computation. Therefore, we do believe that future clinical research based on the proposed multimodal neuro-psycho-physiological features and AI analysis can detect mental health disorders early enough to prevent and reduce the emergence of severe mental illnesses. Such reliable predictors of potential mental health disorders among HCWs due to COVID-19 stressors will be crucial for the mental health of HCWs and maintaining high efficiency and productivity of medical institutions globally.

## Proposed methodology

**Figure 1:**
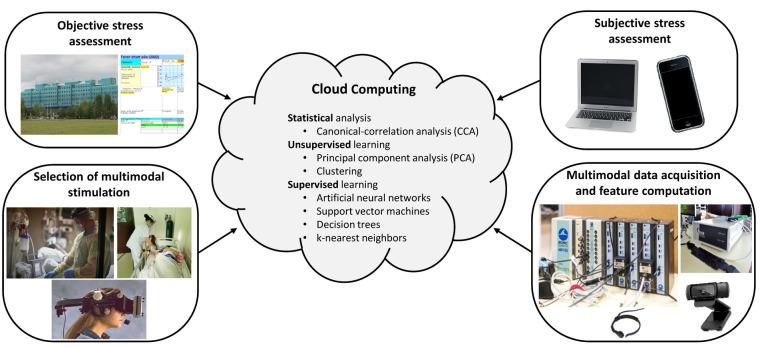
The proposed methodology for prediction of mental health disorders. The illustration was partially assembled from public domain/free sources on Wikipedia and Wikimedia Commons.

The proposed methodology, described in Figure 1 and in the following 5 phases, includes objective assessment of intensity of HCWs’ stressor exposure during the COVID-19 pandemic described in Phase 1, subjective assessment of stress experienced by HCWs during the COVID-19 pandemic based on the specific psychological questionnaire described in Phase 2, distinctive stimulation paradigms designed and developed within Phase 3, computed neuro-physiological features based on stimulation responses in Phase 4, as well as statistical and ML data analysis described in Phase 5.

### Phase 1: Objective stress assessment

Objective assessment of intensity of HCWs’ stressor exposure during the COVID-19 pandemic is based on acquiring information from official hospital archives and clinical records regarding their daily schedules during the COVID-19 pandemic, overtime work, the level of threat they experienced, sick leave, etc. These objective metrics of exposure to stressors are proposed based on analysis and adaptation of different questionnaires that have been used for assessment of stressors in military combat deployment and operation (22-24), as well as stressors in virus outbreaks (25-28). The key aim of this phase is to objectively stratify individual HCWs according to the objective level of stress to which they were exposed during their clinical service, using the information provided by authorized clinical sources rather than by asking individuals to self-report themselves.

### Phase 2: Subjective stress assessment

Subjective assessment of stress experienced by HCWs during their COVID-19 pandemic clinical service is based on the questionnaire that is developed by a selection of the most appropriate items from general-purpose psychological questionnaires used for early recognition of distress, mental health disorder screening, and stress resilience (eg, 29-38), as well as from specific COVID-19 psychological questionnaires (25-28,39). Self-reported subjective peritraumatic reactions represent a valuable complement to objective dimensions of stressful situations collected in Phase 1 when trying to predict chronic mental health disorders, such as PTSD (40). Accordingly, subjective self-reports of individual COVID-19 stress intensity and relevant personality traits will also be used as one of the indicators of potential chronic mental health disorders in comparison with more objective metrics developed in Phase 1.

### Phase 3: Selection of multimodal stimulation

**Figure 2:**
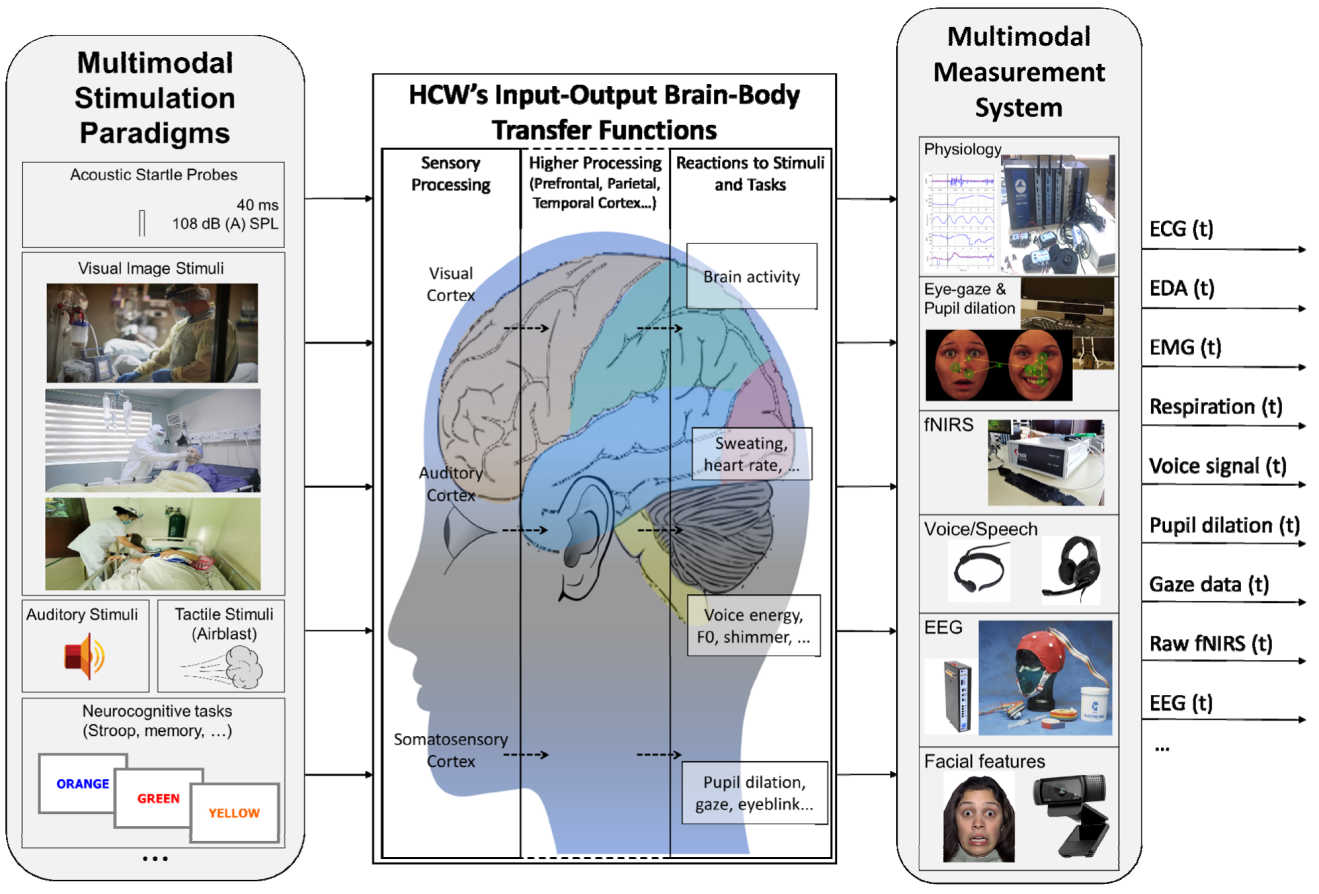
Design and development of multimodal stimulation paradigms for optimal elicitation of specific neuro-psycho-physiological individual reactions; adapted from (21). HCW – health care workers; fNIRS – functional near-infrared spectroscopy; EEG – electroencephalography; ECG – electrocardiography, EMG –electromyography; EDA – electrodermal activity. The illustration was partially assembled from public domain/free sources: *https://publicdomainvectors.org*, *http://www.stockunlimited.com*, *https://commons.wikimedia.org.*

This phase is related to the design and development of appropriate multimodal stimulation paradigms in order to optimally elicit specific neuro-psycho-physiological individual reactions among HCW participants (Figure 2). Accordingly, the appropriate input-output multimodal experimental stimulation paradigms that elicit the specific multimodal features reflecting the impact of stress on the patients’ neuro-psycho-physiological state (21) are usually related to baseline neuro-physiological functioning; well-established generic stressful emotional stimuli, such as different versions of acoustic startle stimuli and airblasts; startle modulation paradigms, such as fear-potentiated and anxiety-potentiated startle (41), and prepulse inhibition; aversive images and sounds semantically related to COVID-19 clinical environments; a variety of cognitive tasks, eg, different versions of Stroop tests (42,43), memory tasks (44), arithmetic tasks (45,46), or verbal fluency tasks (47,48). Developed multimodal stimulation paradigms are administered to the HCWs in a controlled clinical laboratory setting in order to acquire their multimodal neuro-physiological reactions. Acoustic startle stimuli are usually 50 ms immediate rise time broadband noise bursts, with intensity ranging from 95-110 dB[SPL] (49), and are delivered binaurally through headphones. In order to induce laboratory fear, threat, or anxiety by means of predictable and unpredictable aversive events delivery (50), other aversive stimuli can be used, eg, combinations of airblasts to the neck, aversive images on the screen and sounds (51), as well as annoying but not painful electric shocks, eg, 1.5-2.5 mA, 5-ms duration. Existing semantically and emotionally annotated stimuli databases can facilitate efficient and accurate search for optimal aversive audio-visual stimuli to include in the multimodal stimulation paradigms (52,53). Cognitive tasks are usually administered through specifically designed programs that allow response duration and accuracy measurement.

### Phase 4: Multimodal data acquisition and feature computation

**Figure 3:**
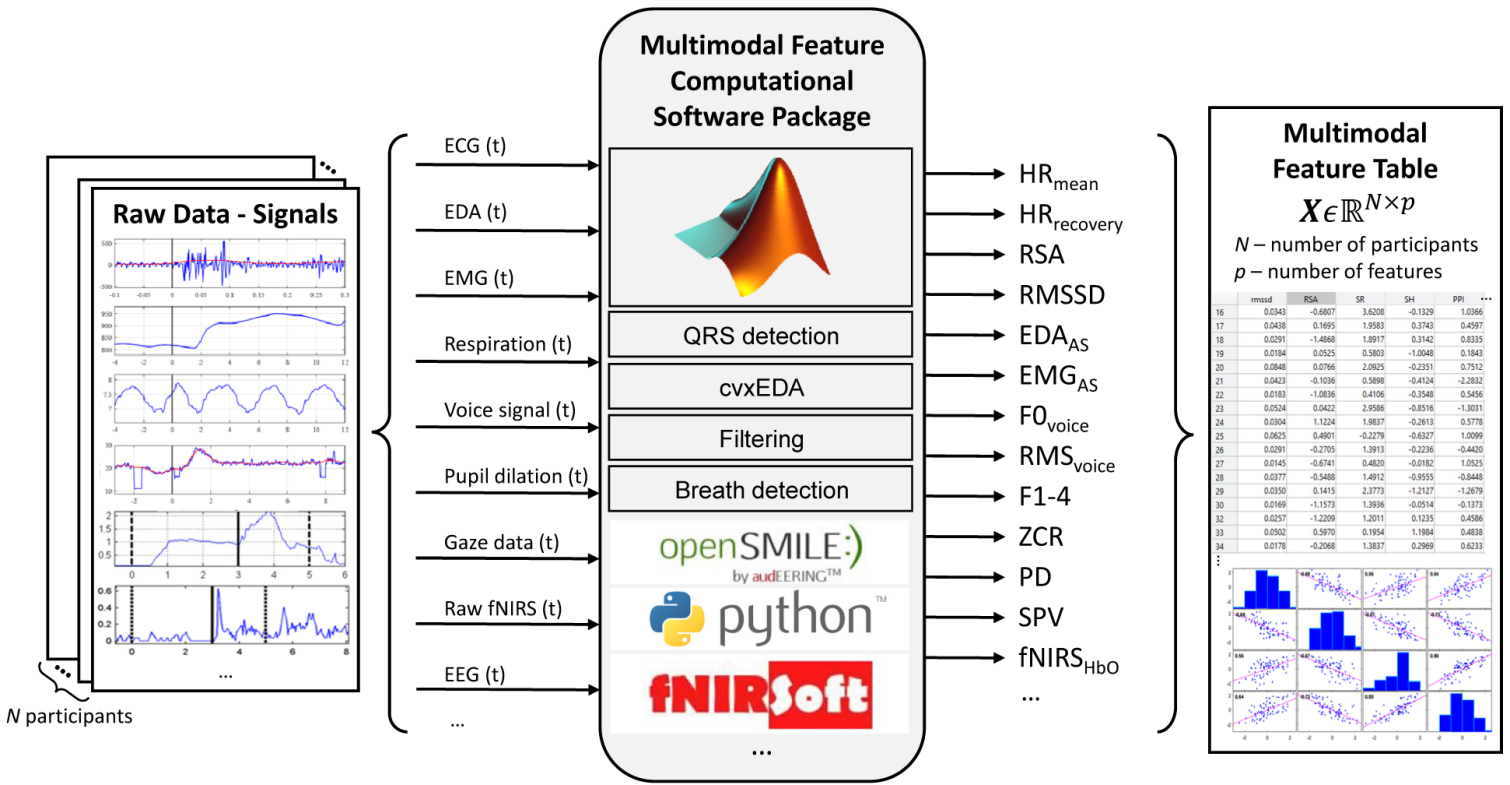
Multimodal data acquisition and feature computation. Illustrated is a subset of features: HR_mean_ – mean heart rate; HR_recovery_ – heart rate recovery; RSA – respiratory sinus arrhythmia; RMSSD – root mean square of successive differences; EDA_AS_ – EDA-based startle response measure; EMG_AS_ – EMG-based startle response measure; F0_voice_ – voice fundamental frequency; RMS_voice_ – voice energy – root mean square; F1-4 – voice formants; ZCR – voice zero-crossing rate; PD – pupil dilation; SPV – saccadic peak velocity; fNIRS_HbO_ – oxygenated hemoglobin.

This phase (Figure 3) is related to the acquisition of multimodal neuro-physiological reactions on stimulation paradigms proposed in the previous phase and computation of corresponding features relevant for prediction of mental health disorders. The proposed methodology is based on state-of-the-art sensors for measurements of the individual’s multimodal neuro-psycho-physiological reactions: functional near-infrared spectroscopy (fNIRS); electroencephalography (EEG); peripheral physiology, ie, electrocardiography (ECG), electromyography (EMG), electrodermal activity (EDA), respiration; speech/acoustic and linguistic reactions; and facial/gesture and oculomotor reactions (54,55). Such measurements, obtained as a response to relevant stimuli described in Phase 3, have the potential to objectivize traditional diagnostic methodology in psychiatry. In our laboratory, the Biopac MP150 system (BIOPAC Systems Inc., Goleta, CA, USA) is used for the acquisition of the neuro-physiological signals. A Gazepoint GP3 HD eye-tracker (Gazepoint, Vancouver, Canada) is used for detection of spontaneous blinks, tracking of changes in pupil dilation, and gaze tracking. A microphone and a webcam are used for collecting speech and gesture data, while the fNIRS Biopac Model 1100 Imager together with the COBI Studio Software (BIOPAC Systems Inc.) is used for brain activation measurements.

After pre-processing of the neuro-physiological signals, ie, obtained inter-beat interval time-series based on the detected QRS complexes in the ECG signal, preprocessed respiratory and EDA data, accordingly filtered EMG data for eyeblink startle response assessment, an array of relevant multimodal features is computed (17,21). These features are elicited and computed according to the relevant research findings related to their associations with specific positive or negative mental health disorder predictors or outcomes, such as stress resilience/vulnerability and other personality traits, distress, anxiety, PTSD, or depression. Therefore, these features are defined and computed in a theory-driven manner. Examples of such features are resting heart rate (56,57) and heart rate variability (HRV) (58,59), respiratory sinus arrhythmia (21,60), HRV-based psychophysiological allostasis (21,58), EMG-based and EDA-based startle reactivity (61), various features related to speech prosody (62), prefrontal cortex activation on various cognitive tasks (43,44), and alpha band-related parietal EEG asymmetry (63). Such integrated multimodal neuro-psycho-physiological prediction of mental health disorders emphasizes the importance of combining different multimodal features in enhancing predictive power of the proposed approach, since any single feature in the assessment and prediction of mental health deterioration is a relatively weak discriminator.

### Phase 5: Data analysis for prediction of mental health disorders

Due to potentially large amounts of highly heterogeneous data, Phase 5 is accomplished using cloud storage and cloud computing resources, as shown in Figure 1. Statistical correlation-based analyses are expected to provide better insight into the neuro-physiological risk markers for the development of chronic stress-related mental health problems affected by the COVID-19 pandemic. Feature selection and classification based on ML, as opposed to statistical methods, would explore more complex interactions between various features in a highly nonlinear manner associated with the inference of risk of HCW individuals for the development of chronic mental health problems. Individuals exhibiting high risk of chronic stress-related mental health problems may urgently need as prevention effective and efficient treatments, using state-of-the-art tools and means of digital psychiatry, such as computerized cognitive behavioral therapy (54) and telepsychiatry, which are efficiently applicable in the early stages of illness (64). A more detailed description of the proposed tools and means of statistical and ML analyses is given in the following section.

## Statistical and machine learning analysis

A data-driven verification of various multimodal neuro-psycho-physiological features extracted in Phase 4 can be obtained by the application of statistical analyses and ML techniques in relation to the objective stress intensity assessment from Phase 1, as well as subjective self-report indicators of experienced stress and relevant psychological traits from Phase 2. Phase 5 can provide valuable insight into neuro-psycho-physiological risk markers for the development of chronic stress-related mental/physical problems in the context of the COVID-19 pandemic, and increase the translational potential of such features. A similar data-mining-based approach has been previously used in the analysis of diagnostic data for differentiating PTSD patients from participants with psychiatric diagnoses other than PTSD (65). This work has demonstrated the applicability of ML for the analysis of PTSD, but only based on the data obtained from structured psychiatric interviews and psychiatric scales, which is analogous just to Phase 2 of the methodology proposed in this article.

In terms of statistical analysis, various correlation analysis approaches can be employed. One example of such methodology is the canonical-correlation analysis (CCA), a technique suitable for investigating the relationships between variables coming from distinct sets, eg, the relationship between variables obtained in Phase 1 and Phase 4, or Phase 2 and Phase 4. In doing so, the CCA will provide interpretable linear combinations of variables from different sets that have a maximum correlation. In order to maximize the statistical power of conclusions, ie, to avoid the large statistical corrections due to conducting numerous exploratory tests for significance of correlation coefficients, several particularly well-founded hypotheses should be defined *a priori*, before the computation of the full correlation matrix. These hypotheses should be those with the most overwhelming evidence from the literature regarding expected pairwise associations between specific objective metrics of the stress intensity exposure, subjective self-report metrics of experienced stress and relevant psychological traits, as well as objectively measured/computed neuro-physiological features. A brief overview of neuro-physiological features with the highest predictive potential according to the research references is given in the description of Phase 4. Additionally, a subset of the obtained data can be used to separate the participants according to specific group memberships, eg, high distress vs low distress. For example, a recent COVID-19-related research paper (28) uses data analogous to our proposed Phase 1 and Phase 2 to define resilience in the face of exposure to a stressor of a given intensity. However, in that work all data were obtained via self-report, while we propose the integration of objectively assessed stressor severity (Phase 1) and self-report data (Phase 2) with the relevant neuro-physiological features (Phase 3 and Phase 4). Accordingly, various regression analyses or even between-group tests can be conducted.

Regarding the application of ML, both unsupervised and supervised learning approaches should be considered. Unsupervised learning approaches, such as principal component analysis, factor analysis, or cluster analysis, do not require labeled data and can help reveal previously undetected patterns in heterogeneous sets of data, and help in the understanding of the relationships between objective stressor severity, self-report assessments, and neuro-psycho-physiological characterization of the participant. For example, a non-classical unsupervised learning approach, based on a brain-inspired spiking neural network (SNN) model trained using EEG data, has provided novel insights into the brain functioning in depression and the effects of mindfulness training on the brain connectivity (66). Such novel unsupervised approaches, based on the spike-timing-dependent plasticity learning rules of the SNN connectivity emerging from complex spatio-temporal brain data, like EEG and fNIRS, which are considered in the proposed methodology, could help reveal and understand early patterns of mental health deterioration in HCWs. When considering labeled data, the main aim of supervised ML, as opposed to statistical methods, is the maximization of classification/prediction accuracy, while sacrificing model explainability and rigorous statistical validation. Accordingly, recent work highlights the need to establish an ML framework in psychiatry that nurtures trustworthiness, focusing on explainability, transparency, and generalizability of the obtained models (11). This approach, regardless of the superior classification/prediction performance, is critical in order for the AI methods to be employed in diagnosis, monitoring, evaluation, and prognosis of mental illness. Supervised learning in the context of the proposed methodology can be formulated both in terms of regression and classification tasks. Neuro-physiological features obtained in Phase 4 can be integrated by a model, eg, support vector machine, random forest, artificial neural network, etc, in the accordingly formulated supervised learning task. For example, data from Phase 4 can be used to model various labels emerging from Phases 1 and 2, such as estimation of objective stressor severity, available from Phase 1; or classification of high vs low distress in HCWs based on the data obtained in Phase 2.

To summarize, technology based on AI and ML can only be as strong as the data the models are trained on, which is particularly important in mental health diagnostics. Currently, for most classification or prediction tasks emerging from the area of mental health, labels are most likely still not quantified well enough to successfully train an algorithm. One possible outcome regarding this labeling issue, as briefly stated in the introductory section, is in data-driven AI technologies helping mental health practitioners re-define mental illnesses more objectively than is currently done in the DSM-5. Additionally, AI can help personalize treatments based on the patient’s unique characteristics. Such unique characteristics are often very subtle and hardly observable by human mental health practitioners. For example, subtle shifts in speech tone or pace can be a sign of mania or depression, and such patterns can now be even more precisely detected by an AI-driven system in comparison to humans. AI can exploit language and speech, among many other available modalities, as one of the critical pathways to detecting patient mental states, especially through mobile devices (67), which should also be regarded as highly important in the context of prediction of mental health disorders induced by the COVID-19 pandemic.

## Conclusion

The proposed methodology for prediction of mental health disorders among HCWs during the ongoing pandemic based on AI-aided data analysis is particularly important since they are a high-risk group for contracting the COVID-19 disease (68) and developing later stress-related symptoms. However, the methodology proposed in this article might be applied generally for all those who were exposed to higher levels of such risks during the COVID-19 pandemic. The main objective of the proposed methodology is to expand subjective metrics as predictors of potential mental health disorders mainly specific for Phase 2 with more objective metrics derived in Phases 1, 3, and 4. The use of neuro-physiological features is expected to provide additional information and increase reliability when identifying particularly at-high-risk individuals. Such efforts are well aligned with the growing literature regarding the application of AI methods in prediction of chronic mental health disorders, which has been initially focused mainly on self-report predictor variables (65,69,70) but has been subsequently extended to speech features (62) and various biomarkers (57,71,72). These efforts should help mental health practitioners make their diagnostics more objectively than currently done in the DSM-5. Acquiring more reliable neuro-psycho-physiological predictors based on objective metrics assessment in early identification of the vulnerable individuals is an important step forward in the prevention of mental health disorders caused by the COVID-19 pandemic. Early identification of mental health disorders based on the proposed methodology as well as early warning indicators and risk factors are prerequisites for on-time prediction and prevention of mental health disorders of the global population, helping clinicians make diagnoses more quickly and accurately, and rapidly providing optimal treatment for patients.

## References

[1] Mohanty A, Kabi A, Mohanty AP (2019). Health problems in healthcare workers: A review.. J Family Med Prim Care.

[2] Huang Y, Zhao N (2020). Generalized anxiety disorder, depressive symptoms and sleep quality during COVID-19 outbreak in China: a web-based cross-sectional survey.. Psychiatry Res.

[3] Lai J, Ma S, Wang Y, Cai Z, Hu J, Wei N (2020). Factors associated with mental health outcomes among health care workers exposed to Coronavirus disease 2019.. JAMA Netw Open.

[4] Lee SM, Kang WS, Cho AR, Kim T, Park JK (2018). Psychological impact of the 2015 MERS outbreak on hospital workers and quarantined hemodialysis patients.. Compr Psychiatry.

[5] Shigemura J, Ursano RJ, Morganstein JC, Kurosawa M, Benedek DM (2020). Public responses to the novel 2019 coronavirus (2019 nCoV) in Japan: mental health consequences and target populations.. Psychiatry Clin Neurosci.

[6] Denollet J, Schiffer AA, Spek V (2010). A general propensity to psychological distress affects cardiovascular outcomes: evidence from research on the type D (distressed) personality profile.. Circ Cardiovasc Qual Outcomes.

[7] Li JB, Yang A, Dou K, Cheung RY. Self-control moderates the association between perceived severity of the coronavirus disease 2019 (COVID-19) and mental health problems among the Chinese public. PsyArXiv. 2020. https://psyarxiv.com/2xadq/10.3390/ijerph17134820 PMC7370094 32635495

[8] Walker FR, Pfingst K, Carnevali L, Sgoifo A, Nalivaiko E (2017). In the search for integrative biomarker of resilience to psychological stress.. Neurosci Biobehav Rev.

[9] American Psychiatric Association. Diagnostic and statistical manual of mental disorders (DSM-5®). American Psychiatric Pub. 2013.

[10] Hariman K, Ventriglio A, Bhugra D (2019). The Future of Digital Psychiatry.. Curr Psychiatry Rep.

[11] Chandler C, Foltz PW, Elvevĺg B (2020). Using Machine Learning in Psychiatry: The Need to Establish a Framework That Nurtures Trustworthiness.. Schizophr Bull.

[12] Ćosić K, Popović S, Šarlija M, Kesedžić I (2020). Impact of human disasters and COVID-19 pandemic on mental health: potential of digital psychiatry.. Psychiatr Danub.

[13] Garcia-Ceja E, Riegler M, Nordgreen T, Jakobsen P, Oedegaard KJ, Třrresen J (2018). Mental health monitoring with multimodal sensing and machine learning: A survey.. Pervasive Mobile Comput.

[14] Graham S, Depp C, Lee EE, Nebeker C, Tu X, Kim HC (2019). Artificial intelligence for mental health and mental illnesses: an overview.. Curr Psychiatry Rep.

[15] Shatte AB, Hutchinson DM, Teague SJ (2019). Machine learning in mental health: a scoping review of methods and applications.. Psychol Med.

[16] Nikfarjam A, Sarker A, O’connor K, Ginn R, Gonzalez G (2015). Pharmacovigilance from social media: mining adverse drug reaction mentions using sequence labeling with word embedding cluster features.. J Am Med Inform Assoc.

[17] Ćosić K, Popović S, Šarlija M, Mijić I, Kokot M, Kesedžić I (2019). New tools and methods in selection of air traffic controllers based on multimodal psychophysiological measurements.. IEEE Access.

[18] Thakur V, Jain A. (2020). COVID 2019-suicides: A global psychological pandemic. Brain Behav Immun.

[19] Dutheil F, Aubert C, Pereira B, Dambrun M, Moustafa F, Mermillod M (2019). Suicide among physicians and health-care workers: A systematic review and meta-analysis.. PLoS One.

[20] Ćosić K, Popović S, Šarlija M, Mijić I, Kokot M, Kesedžić I. Multimodal physiological, voice acoustic, eye gaze and brain imaging features of stress resilience. NATO-Approved Final Report of the Project NATO.MD.SFPP 984829 “Multidisciplinary Metrics for Soldier Resilience Prediction and Training”, 2019.

[21] Ćosić K, Šarlija M, Ivkovic V, Zhang Q, Strangman G, Popović S (2019). Stress resilience assessment based on physiological features in selection of air traffic controllers.. IEEE Access.

[22] Hoge CW, Castro CA, Messer SC, McGurk D, Cotting DI, Koffman RL (2004). Combat duty in Iraq and Afghanistan, mental health problems, and barriers to care.. N Engl J Med.

[23] Guyker WM, Donnelly K, Donnelly JP, Dunnam M, Warner GC, Kittleson J (2013). Dimensionality, reliability, and validity of the combat experiences scale.. Mil Med.

[24] Skipper LD, Forsten RD, Kim EH, Wilk JD, Hoge CW (2014). Relationship of combat experiences and alcohol misuse among US special operations soldiers.. Mil Med.

[25] Khalid I, Qabajah MR, Barnard AG, Qushmaq IA (2016). Healthcare workers emotions, perceived stressors and coping strategies during a MERS-CoV outbreak.. Clin Med Res.

[26] Abolfotouh MA, AlQarni AA, Al-Ghamdi SM, Salam M, Al-Assiri MH, Balkhy HH (2017). An assessment of the level of concern among hospital-based health-care workers regarding MERS outbreaks in Saudi Arabia.. BMC Infect Dis.

[27] Wong TY, Koh GC, Cheong SK, Lee HY, Fong YT, Sundram M (2008). Concerns, perceived impact and preparedness in an avian influenza pandemic–a comparative study between healthcare workers in primary and tertiary care.. Ann Acad Med Singapore.

[28] Veer IM, Riepenhausen A, Zerban M, Wackerhagen C, Engen H, Puhlmann L, et al. Mental resilience in the Corona lockdown: First empirical insights from Europe. PsyArxiv. 2020. https://psyarxiv.com/4z62t/

[29] Andrews G, Slade T (2001). Interpreting scores on the Kessler psychological distress scale (K10).. Aust N Z J Public Health.

[30] Bracha HS, Williams AE, Haynes SN, Kubany ES, Ralston TC, Yamashita JM (2004). The STRS (shortness of breath, tremulousness, racing heart, and sweating): A brief checklist for acute distress with panic-like autonomic indicators; development and factor structure.. Ann Gen Hosp Psychiatry.

[31] Lins L, Carvalho FM (2016). SF-36 total score as a single measure of health-related quality of life: Scoping review.. SAGE Open Med.

[32] Roberti JW, Harrington LN, Storch EA (2006). Further psychometric support for the 10-item version of the perceived stress scale.. J Coll Couns.

[33] Weiss DS, Marmar CR. The Impact of Event Scale - Revised. In: Wilson J, Keane TM, eds. Assessing psychological trauma and PTSD. New York: Guilford; 1996. p. 399-411.

[34] Sokołowski A, Dragan WŁ (2017). New empirical evidence on the validity and the reliability of the early life stress questionnaire in a polish sample.. Front Psychol.

[35] Connor KM, Davidson JR (2003). Development of a new resilience scale: The Connor-Davidson resilience scale (CD-RISC).. Depress Anxiety.

[36] Judge TA, Erez A, Bono JE, Thoresen CJ (2003). The core self-evaluations scale: Development of a measure.. Person Psychol.

[37] Deacon BJ, Abramowitz JS, Woods CM, Tolin DF (2003). The Anxiety Sensitivity Index-Revised: psychometric properties and factor structure in two nonclinical samples.. Behav Res Ther.

[38] Spielberger CD, Vagg PR (1984). Psychometric properties of the STAI: a reply to Ramanaiah, Franzen, and Schill.. J Pers Assess.

[39] Ehrenreich-May J. Fear of Illness and Virus Evaluation (FIVE) – Adult Report Form. 2020.

[40] Bernat JA, Ronfeldt HM, Calhoun KS, Arias I (1998). Prevalence of traumatic events and peritraumatic predictors of posttraumatic stress symptoms in a nonclinical sample of college students.. J Trauma Stress.

[41] Jovanovic T, Duncan EJ, Kaye J, Garza K, Norrholm SD, Inslicht SS (2020). Psychophysiological treatment outcomes: Corticotropin-releasing factor type 1 receptor antagonist increases inhibition of fear-potentiated startle in PTSD patients.. Psychophysiology.

[42] Armstrong CM, Reger GM, Edwards J, Rizzo AA, Courtney CG, Parsons TD (2013). Validity of the Virtual Reality Stroop Task (VRST) in active duty military.. J Clin Exp Neuropsychol.

[43] Yennu A, Tian F, Smith-Osborne A, Gatchel RJ, Woon FL, Liu H (2016). Prefrontal responses to Stroop tasks in subjects with post-traumatic stress disorder assessed by functional near infrared spectroscopy.. Sci Rep.

[44] Tian F, Yennu A, Smith-Osborne A, Gonzalez-Lima F, North CS, Liu H (2014). Prefrontal responses to digit span memory phases in patients with post-traumatic stress disorder (PTSD): a functional near infrared spectroscopy study.. Neuroimage Clin.

[45] Brugnera A, Zarbo C, Adorni R, Compare A, Sakatani K (2017). Cortical and autonomic stress responses in adults with high versus low levels of trait anxiety: A pilot study.. Adv Exp Med Biol.

[46] Adorni R, Brugnera A, Gatti A, Tasca GA, Sakatani K, Compare A (2018). Psychophysiological responses to stress related to anxiety in healthy aging: A near-infrared spectroscopy (NIRS) study.. J Psychophysiol.

[47] Nishimura Y, Tanii H, Hara N, Inoue K, Kaiya H, Nishida A (2009). Relationship between the prefrontal function during a cognitive task and the severity of the symptoms in patients with panic disorder: a multi-channel NIRS study.. Psychiatry Res Neuroimaging.

[48] Yokoyama C, Kaiya H, Kumano H, Kinou M, Umekage T, Yasuda S (2015). Dysfunction of ventrolateral prefrontal cortex underlying social anxiety disorder: A multi-channel NIRS study.. Neuroimage Clin.

[49] Vaidyanathan U, Patrick CJ, Cuthbert BN (2009). Linking dimensional models of internalizing psychopathology to neurobiological systems: Affect-modulated startle as an indicator of fear and distress disorders and affiliated traits.. Psychol Bull.

[50] Schmitz A, Grillon C (2012). Assessing fear and anxiety in humans using the threat of predictable and unpredictable aversive events (the NPU-threat test).. Nat Protoc.

[51] Ćosić K, Popović S, Kukolja D, Dropuljić B, Ivanec D, Tonković M (2016). Multimodal analysis of startle type responses.. Comput Methods Programs Biomed.

[52] Horvat M. Generiranje multimedijskih pobuda temeljeno na ontološkom afektivnom i semantičkom označavanju. Doctoral dissertation, University of Zagreb. Faculty of Electrical Engineering and Computing, 2013.

[53] Horvat M, Bogunović N, Ćosić K (2014). STIMONT: a core ontology for multimedia stimuli description.. Multimedia Tools Appl.

[54] Ćosić K, Popović S, Horvat M, Kukolja D, Dropuljić B, Kovač B (2013). Computer-aided psychotherapy based on multimodal elicitation, estimation and regulation of emotion. Psychiatr Danub.

[55] Strangman GE, Ivkovic V, Zhang Q (2018). Wearable brain imaging with multimodal physiological monitoring.. J Appl Physiol.

[56] Shalev AY, Sahar T, Freedman S, Peri T, Glick N, Brandes D (1998). A prospective study of heart rate response following trauma and the subsequent development of posttraumatic stress disorder.. Arch Gen Psychiatry.

[57] Dean KR, Hammamieh R, Mellon SH, Abu-Amara D, Flory JD, Guffanti G (2019). Multi-omic biomarker identification and validation for diagnosing warzone-related post-traumatic stress disorder.. Mol Psychiatry.

[58] Souza GGL, Magalhaes LN, Da Cruz TAR, Mendonça-De-Souza ACF, Duarte AFA, Fischer NL (2013). Resting vagal control and resilience as predictors of cardiovascular allostasis in peacekeepers.. Stress.

[59] Carnevali L, Thayer JF, Brosschot JF, Ottaviani C (2018). Heart rate variability mediates the link between rumination and depressive symptoms: A longitudinal study.. Int J Psychophysiol.

[60] Shader TM, Gatzke-Kopp LM, Crowell SE, Reid MJ, Thayer JF, Vasey MW (2018). Quantifying respiratory sinus arrhythmia: Effects of misspecifying breathing frequencies across development. ‎. Dev Psychopathol.

[61] Shalev AY, Peri T, Brandes D, Freedman S, Orr SP, Pitman RK (2000). Auditory startle response in trauma survivors with posttraumatic stress disorder: a prospective study.. Am J Psychiatry.

[62] Marmar CR, Brown AD, Qian M, Laska E, Siegel C, Li M (2019). Speech-based markers for posttraumatic stress disorder in US veterans.. Depress Anxiety.

[63] Butt M, Espinal E, Aupperle RL, Nikulina V, Stewart JL (2019). The electrical aftermath: brain signals of posttraumatic stress disorder filtered through a clinical lens.. Front Psychiatry.

[64] Sijbrandij M, Olff M, Reitsma JB, Carlier IV, de Vries MH, Gersons BP (2007). Treatment of acute posttraumatic stress disorder with brief cognitive behavioral therapy: a randomized controlled trial.. Am J Psychiatry.

[65] Marinić I, Supek F, Kovačić Z, Rukavina L, Jendričko T, Kozarić-Kovačić D (2007). Posttraumatic stress disorder: diagnostic data analysis by data mining methodology.. Croat Med J.

[66] Doborjeh Z, Doborjeh M, Taylor T, Kasabov N, Wang GY, Siegert R (2019). Spiking neural network modelling approach reveals how mindfulness training rewires the brain.. Sci Rep.

[67] Foltz PW, Rosenstein M, Elvevĺg B (2016). Detecting clinically significant events through automated language analysis: Quo imus?. NPJ Schizophr.

[68] Čivljak R, Markotić A, Kuzman I (2020). The third coronavirus epidemic in the third millennium: what’s next?. Croat Med J.

[69] Galatzer-Levy IR, Karstoft KI, Statnikov A, Shalev AY (2014). Quantitative forecasting of PTSD from early trauma responses: A machine learning application.. J Psychiatr Res.

[70] Karstoft KI, Galatzer-Levy IR, Statnikov A, Li Z, Shalev AY (2015). Bridging a translational gap: using machine learning to improve the prediction of PTSD.. BMC Psychiatry.

[71] Galatzer-Levy IR, Ma S, Statnikov A, Yehuda R, Shalev AY (2017). Utilization of machine learning for prediction of post-traumatic stress: a re-examination of cortisol in the prediction and pathways to non-remitting PTSD.. Transl Psychiatry.

[72] Schultebraucks K, Shalev A, Michopoulos V, Stevens J, Jovanovic T, Bonanno G (2020). A generalized predictive algorithm of posttraumatic stress development following emergency department admission using biological markers routinely collected from electronic medical records.. Biol Psychiatry.

